# Assessing anxiety during the COVID-19 delta epidemic: Validation of the Chinese coronavirus anxiety scale

**DOI:** 10.3389/fpsyg.2022.981121

**Published:** 2022-09-14

**Authors:** Qiaoping Lian, Lu Xia, Daxing Wu

**Affiliations:** ^1^Medical Psychological Center, The Second Xiangya Hospital, Central South University, Changsha, Hunan, China; ^2^Medical Psychological Institute of Central South University, Changsha, Hunan, China; ^3^National Clinical Research Center on Mental Disorders, Changsha, Hunan, China

**Keywords:** COVID-19 delta pandemic, coronavirus anxiety scale, anxiety, validity, reliability

## Abstract

The study aimed to examine the psychometric properties of the coronavirus anxiety scale (CAS) during the coronavirus disease 2019 (COVID-19) delta epidemic. A total of 2,116 participants on the Chinese mainland completed the online survey. We employed exploratory factor analysis (EFA) and confirmatory factor analysis (CFA) to investigate the factor structure. The findings showed that the one-factor model of the CAS Chinese version fitted perfectly with the data. The multigroup CFAs showed the measurement invariance across gender and age groups (18–29 and 30–68). We also examined the CAS’s internal consistency and convergent and concurrent validity. The results demonstrated that the one-factor model had good reliability and convergent and concurrent validity. Overall, according to our findings, the CAS Chinese version was reliable for measuring coronavirus anxiety during the COVID-19 delta outbreak.

## Introduction

The epidemic of coronavirus illness 2019 (COVID-19) became a global health emergency, and the virus that caused COVID-19 evolved. The World Health Organization had so far identified five SARS-CoV-2 variants as being of concern (Tracking SARS-CoV-2 Variants, 2020). In May 2021, the B.1.617.2 (delta) variant was designated a variant of concern by the World Health Organization (Tracking SARS-CoV-2 Variants, 2020). The variant was characterized by higher transmission rates than other variants (Dougherty et al., 2021), high pathogenicity, high viral load, and a short incubation period (Chen X. et al., 2021). The delta variant caused a resurgence in China from July to August 2021. It led to new spikes in China, almost equal to the country’s total number in the previous 5 months (China goes all out to contain the Delta outbreak, 2021).

The complexity and uncertainty of the epidemic caused human physical and mental problems. To prevent the rapid spread of the virus, some enforcement rules or guidelines, such as “social isolation,” “international travel restrictions,” “mask-wearing,” and “quarantine,” were taken by countries, territories, and areas [WHO Coronavirus (COVID-19) Dashboard, 2022]. Although these rules or guidelines had worked in preventing the spread of COVID-19, they also induced psychological problems. Plenty of studies reported negative psychological effects due to the pandemic, such as depression, anxiety (Hu et al., 2020), worry (Tull et al., 2020), fear, and posttraumatic stress symptoms (Brooks et al., 2020). Stress, depression, and anxiety were the primary prevalent psychological problems during the early period in China (Bareeqa et al., 2021). A review showed that the anxiety prevalence was 27.3% among the general population (Pashazadeh Kan et al., 2021). It was worth noting that the resurgence of COVID-19 exacerbated the psychological impacts due to the pandemic (Broche-Pérez et al., 2021). Additionally, the second wave of the epidemic also affected human’ psychological health (Lizana and Lera, 2022). The virus that caused COVID-19 changed over time, and researchers found trends related to anxiety increased in India when the delta variant appeared (Awijen et al., 2022).

During the COVID-19 pandemic, Lee (2020) created the coronavirus anxiety scale (CAS) to test for coronavirus anxiety. A score of less than nine on the CAS indicated dysfunctional anxiety (Lee, 2020). According to the Early Career Psychiatrist-World Psychiatric Association, the CAS was added to the COVID-19 psychological health care toolkit to help identify people who needed psychological health care during the pandemic (Adiukwu et al., 2020). The single-factor model had good reliabilities and demonstrated no gender differences (Chen J. et al., 2021; Lieven, 2021). Recent studies found that the five-item model sometimes indicated a poor model fit. Some studies suggested that using modification indices would improve the model fit (Magano et al., 2021; Broche-Pérez et al., 2022), so the error covariance correlations were added in their studies, like between item 1 and item 3 (Karaahmet et al., 2022), as well as between item 4 and item 5 (Mora-Magaña et al., 2022). The other studies suggested a four-item model that excluded item 4 or item 5. The four-item model was proved in all 12 Latin American countries after excluding item 5 (Caycho-Rodríguez et al., 2022). Both two models showed good model fit. This scale had already been adopted in many countries such as Turkey (Işik et al., 2022), Cuba (Broche-Pérez et al., 2022), Mexico (Mora-Magaña et al., 2022), and China (Chen J. et al., 2021). A cross-cultural online survey showed reliable global validation across 25 countries across six continents (Lieven, 2021). To further assess the CAS’s psychometric properties, researchers validated it in various samples, such as the college sample (Serpas and Ignacio, 2021), the healthcare professional sample (Mora-Magaña et al., 2022), and the patients with preexisting psychiatric disorder sample (Karaahmet et al., 2022). There was a need for more evidence to assess the applicability of existing validity evidence in new situations (Fairchild et al., 2005). According to Gjersing et al. (2010), the validated instruments some time ago might no longer be valid in the present due to ongoing changes in society. It was in line with the view that the virus that caused COVID-19 changed over time. Although the CAS has been widely used, its psychometric properties were not discussed at different times. Chen J. et al. (2021) also suggested that future studies to track the changes were warranted because of the fluctuations in psychiatric symptoms during the pandemic. Therefore, measuring the CAS validation was essential during the COVID-19 delta epidemic. Meanwhile, there was only the Traditional Chinese Characters language version, and a Simplified Chinese Characters of Chinese mainland validation of Lee’s CAS was not yet available. Thus, we translated and tested the CAS Chinese version in the Chinese mainland sample to provide a helpful screening tool for coronavirus anxiety during the COVID-19 delta epidemic.

The objective of the current study was to assess the CAS’ psychometric characteristics among the sample of Chinese mainland residents during the COVID-19 delta outbreak. Firstly, we investigated its factor structure. We hypothesized that the data would be well-fitted by the one-factor model. We also hypothesized that the instrument was equivalent across gender and age based on the previous studies. Secondly, we calculated the internal consistency values to assess the reliability of the CAS. We hypothesized that the CAS would be a reliable tool to measure coronavirus anxiety during the outbreak. Thirdly, the convergent validity was evaluated using composite reliability (CR) and average variance extracted (AVE). Finally, about concurrent validity, the relationships between the Fear of COVID-19 Scale (FCV-19S), Depression anxiety stress scales (DASS-21), and the CAS were examined. We hypothesized that the CAS had good convergent and concurrent validity, which meant the CAS would be a psychometrically sound tool for assessing the anxiety related to COVID-19.

## Materials and methods

### Participants and procedure

Over 1 week in August 2021, we conducted a cross-sectional survey online through the WeChat public platform on the Chinese mainland. All participants might see this survey and answer the questionnaire using WeChat. Before answering the questionnaire, each participant’s electronic informed consent was collected. This online survey was entirely voluntary and non-commercial. Without giving a reason, respondents were free to leave the survey whenever they wanted. Participants agreed to the online informed consent statement and completed the questionnaires. After completing the scales, every participant would receive a reward containing an individual report and 1–3 CNY. The ethics committee of the Second Xiangya Hospital of Central South University approved the research.

A total of 5,417 questionnaires were collected. To ensure data quality, questionnaires were invalid if they meet the exclusion criteria. The exclusion criteria included: (1) time for each item completion less than 2 s; (2) the questionnaires were consecutive identical item responses. Finally, 2,116 questionnaires were included in the final analysis.

### Measures

#### The coronavirus anxiety scale

The CAS was a self-report tool created to estimate the levels of COVID-19-related anxiety (Lee, 2020). It consisted of 5 items. It was a short mental health screener to estimate present anxiety over the prior 2 weeks. Items were scored on a 5-point Likert scale.

The steps of the translation of the Chinese version were as follows. Firstly, the preliminary translation of the CAS from English to Chinese was done by two native Chinese-speaking researchers with high English proficiency from the research team. Subsequently, the version of the preliminary translation was reviewed by a clinical psychologist to verify the appropriateness of all meanings and expressions. Modifications were made after the group reached a consensus. The final step involved translating the CAS Chinese version back into English. We compared the back-translated version and the original version to ensure accuracy. A research team of 3 researchers reviewed and checked the translation. Finally, the CAS Chinese version was confirmed.

#### The fear of COVID-19 scale

The fear of COVID-19 Scale (FCV-19S) was an instrument designed to measure the levels of COVID-19-related fear (Ahorsu et al., 2020). It consisted of 7 items. Items were scored on a 5-point Likert scale. The higher scores on this scale indicated greater fear of COVID-19. It has been validated in China (Chi et al., 2022). In the current study, Cronbach’s alpha was 0.95.

#### Depression anxiety stress scales

The Depression anxiety stress scales (DASS-21) was an instrument designed to measure the past week’s experience of depression, anxiety, and stress (Henry and Crawford, 2005). It consisted of 21 items and included depression, anxiety, and stress subscales. Each subscale included seven items. Items were scored on a 4-point Likert scale. The scale has been validated in China (Wang et al., 2016). In the current study, the Cronbach alpha coefficient of the DASS-21 subscales was determined to be 0.95 (depression), 0.94 (anxiety), and 0.94 (stress).

## Statistical analysis

Utilizing Amos 23.0 and SPSS 22.0, data were analyzed. Firstly, we employed descriptive statistics to evaluate the participant characteristics (i.e., frequency, percentage, mean, and standard deviation). Secondly, to assess the factor structure of the CAS Chinese version, we conducted EFA and CFA in the current sample. The total number of data (*n* = 2,116) were randomly divided into two samples, hereafter referred to as Sample 1 (*n*_1_ = 1,058) and Sample 2 (*n*_2_ = 1,058). EFA was conducted in Sample 1 and CFA in Sample 2. Principal component analysis (PCA) with varimax rotation was used to perform EFA. The Kaiser–Meyer–Olkin (KMO) tests were used to evaluate the appropriateness of the data in the first stage. In EFA, the KMO statistic ranged from 0 to 1. A value near 1 indicated the more robust the correlation between the variables, which implied that they were suitable for factor analysis. The second step was the factor extraction process, which was the substance step in conducting factor analysis. PCA was used to analyze the data to receive the minimum number of dimensions needed to represent the current data set. The eigenvalues in this stage allowed us to see how the generated factors were determined. We extracted factors with eigenvalues larger than one. Factor loading values of 0.4 or higher were regarded as satisfactory. Goodness-of-fit index (GFI), comparative fit index (CFI), Tucker–Lewis index (TLI), root-mean-square-error of approximation (RMSEA), and standardized root-mean residual (SRMR) were used to assess the model fit. Values of CFI, GFI, and TLI ≥ 0.95 (Hu and Bentler, 1999), RMSEA between 0.06 and 0.08, and SRMR ≤0.08 (Schreiber et al., 2006) suggested a good model fit.

Subsequently, we performed multigroup CFAs to assess the measurement invariance of the Chinese version of the CAS across gender and age groups. We examined the measurement invariance of the CAS in a series of multigroup CFAs in a four-step procedure that imposed increasingly stringent equality constraints on model parameters (i.e., male vs. female; 18–29 vs. 30 years and older) across groups. The four measurement invariance steps were: (1) configural, equivalence of model form; (2) metric, equivalence of factor loadings; (3) scalar, equivalence of item intercepts or thresholds; and (4) residual, the equivalence of items’ residuals or unique variances (Putnick and Bornstein, 2016). In view that the Chi-square indices were sensitive to the size of the sample, we focused on comparing the CFI, RMSEA, and SRMR indices. ΔCFI values were less than <0.010, ΔRMSEA values were less than <0.015, and ΔSRMR values were less than ≤0.010. These differences indicated that the models were measurement invariant between groups (Chen Fang, 2007).

Thirdly, we evaluated corrected item-total correlation (accepted value ≥0.30) as well as internal consistency reliabilities (Cronbach’s alpha, McDonald’s omega, and split-half reliability; accepted value ≥0.70).

Finally, according to Fornell and Larcker (1981), the convergent validity of an instrument was determined by examining two variables, the AVE of the latent variable and the CR of the measure. The convergent validity was measured using AVE and CR. Convergent validity could be considered sufficient when AVE values were ≥ 0.50 and CR values were ≥ 0.70. Concurrent validity was estimated by computing the correlation coefficient between the CAS and other correlated scales (FCV-19S and DASS-21).

## Results

### Demographic variable

We summarized the participants’ characteristics in Table 1. Respondents aged from 18 to 68 (*M* = 31.21, SD = 9.51), with females accounting for 59.0% of total participants. Most of the participants got vaccinated (67.6%). The majority of the sample had a monthly income of RMB 5,000 to 9,999 (42.9%).

**Table 1:** Sample characteristics.

Characteristic	Variable	*M*	SD
Age		31.21	9.51
		*Count*	*Percent %*
Gender	Male	868	41.0
	Female	1,248	59.0
Education level	Junior school and below	288	13.6
	Senior school	738	34.9
	Bachelor	982	46.4
	Master and above	108	5.1
Marital status	Single	866	40.9
	Married	1,204	56.9
	Divorced	37	1.8
	Widowed	9	0.4
Monthly income level (CNY)	2,000 or less	398	18.8
	2,000–4,999	623	29.4
	5,000–9,999	908	42.9
	10,000 or more	187	8.9
Occupation	Healthcare workers^a^	507	24.0
	Enterprise or institution workers^b^	600	28.3
	Teachers or students^c^	514	24.3
	Others^d^	495	23.4
Vaccination	Not vaccinated	686	32.4
	Vaccinated	1,430	67.6

a Included doctors, nurses, disease control staff, medical departmental managers, and psychological counselors.

b Included government personnel, community staff, volunteers, social workers, and policies.

c Included teachers or students from universities, middle schools, or elementary schools.

d Included freelancers, retirees, and other relevant staff. *M* = mean, *SD* = Standard deviation.

### Factor analysis

First, we performed EFA to explore the factor structure of CAS. The sampling adequacy of KMO was 0.894, and Bartlett’s test of sphericity was <0.001, which indicated sufficient support for the factor solution. We used PCA as the extraction method, and the rotation method was varimax rotation. The PCA resulted in a single factor explaining 81.80% of the total variance. Rotated factor loadings exceeding 0.40 ranged from 0.86 (item 1) to 0.93 (item 4). The factors were no cross-loadings.

**Figure 1:**
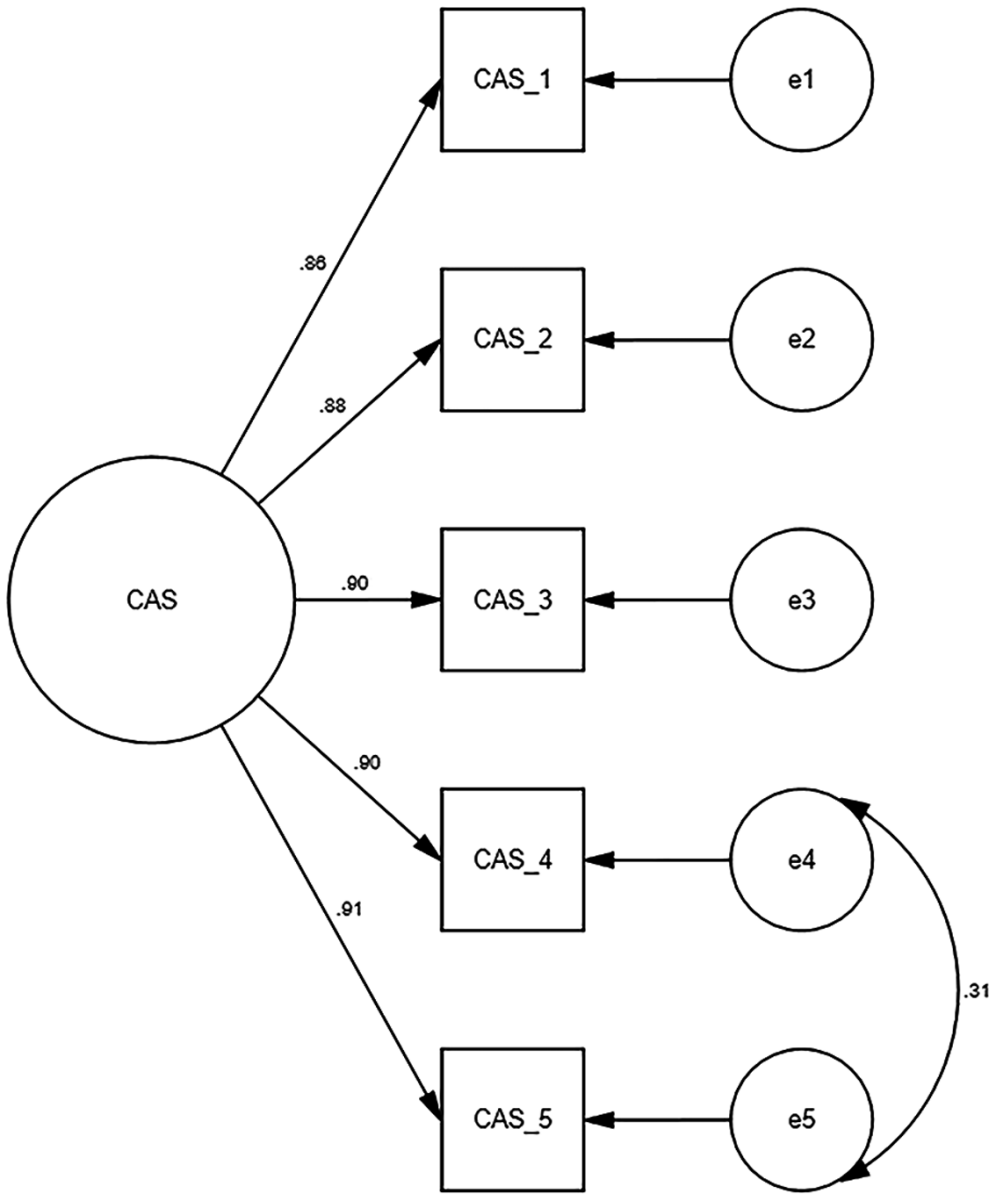
Confirmatory factor analysis of the coronavirus anxiety scale.

Secondly, we further evaluated the one-factor structure in the CFA. The results showed that the single-factor yielded excellent fit for the following indices (GFI = 0.975, CFI = 0.989, TLI = 0.978, and SRMR = 0.013), but not the RMSEA (RMSEA = 0.106). In our study (Figure 1), an error covariance correlation was allowed between item 4 (“*I lost interest in eating when I thought about or was exposed to information about the coronavirus*”) and item 5 (“*I felt nauseous or had stomach problems when I thought about or was exposed to information about the coronavirus*”). The new one showed a good model fit (GFI = 0.993, CFI = 0.997, TLI = 0.994, RMSEA = 0.057, and SRMR = 0.006) after adjusting for the error between items 4 and 5.

### Measurement invariances by gender and age

Table 2 showed that the invariance model fitted well for data of different genders at configural, metric, scalar, and residual levels (CFI = 0.992 to 0.994, RMSEA = 0.041 to 0.060, SRMR = 0.013 to 0.022). The comparison of the metric and configuration model showed that scaled ΔCFI = 0.000, ΔRMSEA = − 0.008, ΔSRMR = 0.000, which indicated the equivalent metric invariance across gender. We found the similar results when restricting item intercepts (scalar model: ΔCFI = 0.000; ΔRMSEA = − 0.010; ΔSRMR = 0.000) and residuals (residual model: ΔCFI = − 0.002; ΔRMSEA = − 0.001; ΔSRMR = 0.009).

**Table 2:** Measurement invariances of the coronavirus anxiety scale across gender and age groups.

Model	*χ* ^2^	*df*	Δ*χ*^2^	value of *p*	CFI	ΔCFI	RMSEA	ΔRMSEA	SRMR	ΔSRMR
Gender (male vs. female)										
Configural	38.124	8		<0.001	0.994		0.060		0.013	
Metric	45.894	12	7.770	<0.001	0.994	0.000	0.052	−0.008	0.013	0.000
Scalar	49.008	17	3.114	<0.001	0.994	0.000	0.042	−0.010	0.013	0.000
Residual	66.708	24	17.700	<0.001	0.992	−0.002	0.041	−0.001	0.022	0.009
Age (18–29 vs. 30–68)										
Configural	48.621	8		<0.001	0.993		0.069		0.010	
Metric	55.944	12	7.323	<0.001	0.992	−0.001	0.059	−0.010	0.011	0.001
Scalar	61.253	17	5.309	<0.001	0.992	0.000	0.050	−0.009	0.011	0.000
Residual	87.871	24	26.618	<0.001	0.988	−0.004	0.050	0.000	0.013	0.002

*df*, degrees of freedom; CFI, Comparative fit index; RMSEA, Root-mean-square-error of approximation; SRMR, Standardized root-mean square residual; Δ, Change.

Table 2 also showed that invariance model fitted the data of 18 to 29 and 30 to 68 years old respondents well at configural, metric, scalar, and residual levels (CFI = 0.988–0.993, RMSEA = 0.050–0.069, and SRMR = 0.010–0.013). The comparison of the metric and configuration model showed that scaled ΔCFI = −0.001, ΔRMSEA = −0.010, and ΔSRMR = 0.001, which indicated the equivalent metric invariance across age. We found the similar results when restricting item intercepts (scalar model: ΔCFI = 0.000; ΔRMSEA = −0.009; and ΔSRMR = 0.000) and residuals (residual model: ΔCFI = −0.004; ΔRMSEA = 0.000; and ΔSRMR = 0.002). These results demonstrated the measurement invariance of the Chinese version of CAS concerning gender and age.

### Internal consistency reliability

The internal consistency of the CAS Chinese version was calculated using Cronbach’s alpha, McDonald’s omega, and Spearman–Brown coefficients. The Cronbach’s alpha coefficient, McDonald’s omega coefficient, and split-half reliability for the total score were 0.95, 0.95, and 0.94, respectively, which all exceeded the 0.70 level. These findings demonstrated the CAS Chinese version’s excellent reliability. Each item’s mean and standard deviation were displayed in Table 3 and ranged from 0.31 (0.81) to 0.43 (0.84). Good corrected item-total correlations were also shown in Table 3, with items varying from 0.81 (item 1) to 0.89 (item 5).

**Table 3:** Descriptive and psychometric properties of the coronavirus anxiety scale at the item level.

Item	*M*	SD	Corrected item-total correlation	Squared-multiple correlation	Cronbach’s alpha if the item deleted
CAS_1	0.34	0.82	0.81	0.66	0.94
CAS_2	0.43	0.84	0.84	0.71	0.94
CAS_3	0.39	0.87	0.87	0.75	0.93
CAS_4	0.37	0.82	0.88	0.81	0.93
CAS_5	0.31	0.81	0.89	0.82	0.93

CAS, Coronavirus anxiety scale; *M*, Mean; *SD*, Standard deviation.

### Convergent validity and concurrent validity

As shown in Figure 1, the factor loadings of the CAS varied from 0.86 (item 1) to 0.91 (item 5). The factor loading of each item was greater than 0.70. The AVE value was 0.793 exceeding 0.50, and the CR value was 0.950 exceeding 0.70. These findings demonstrated the CAS had good convergent validity. Regarding the concurrent validity, the CAS total scores were positively correlated with FCV-19S (*r* = 0.52), DASS_21 depression subscale (*r* = 0.51), DASS_21 anxiety subscale (*r* = 0.56), and DASS_21 stress subscale (*r* = 0.53). As shown in Table 4, the results provided an evidence of criterion validity.

**Table 4:** Pearson’s correlations between the FCV-19S, the DASS-21, and the CAS.

	CAS	FCV-19S	DASS_D	DASS_A	DASS_S
CAS	1				
FCV-19S	0.52^**^	1			
DASS_D	0.51^**^	0.53^**^	1		
DASS_A	0.56^**^	0.58^**^	0.91^**^	1	
DASS_S	0.53^**^	0.57^**^	0.92^**^	0.92^**^	1

** *p* < 0.01.

CAS, Coronavirus anxiety scale; FCV-19S, The Fear of COVID-19 Scale; DASS-21, Depression anxiety stress scales; DASS_D, DASS-21 depression subscale; DASS_A, DASS-21 anxiety subscale; and DASS_S, DASS-21 stress subscale.

## Discussion

The current study’s objective was to validate the CAS in the sample from the Chinese mainland during the COVID-19 delta pandemic. After EFA, the single-factor solution yielded a good model fit in CFA. We showed the CAS possessed stability across gender and age (18–29 and 30–68) groups. The findings also demonstrated excellent internal consistency and convergent and concurrent validity, which indicated the Chinese version of the CAS was a reliable tool during the COVID-19 delta epidemic.

Through the EFA, the PCA of five items supported the single-factor model of the CAS. The results of CFA also supported the single-factor structure. These results were in line with the previous studies (Lee, 2020; Lieven, 2021; Karaahmet et al., 2022), which indicated one latent construct for the CAS. Consistent with other research (Padovan-Neto et al., 2021), the RMSEA did not fit the model well in our research. The possible reason for the poor fitting model might be that the *df* was too small. The theoretical analysis found that the values of the RMSEA in small *df* models might falsely indicate a poorly fit model (Kenny et al., 2015). Some studies used modification indices to improve the model fit (Magano et al., 2021; Broche-Pérez et al., 2022). Mora-Magaña et al. (2022) validated this instrument in Mexican healthcare professionals, and they achieved a better model after adjusting the error of item 4 (“*I lost interest in eating when I thought about or was exposed to information about the coronavirus*”) and item 5 (“*I felt nauseous or had stomach problems when I thought about or was exposed to information about the coronavirus*”). By adjusting for the error of item 4 and item 5 in the same way, our results also showed an excellent model fit. That was might because item 4 and item 5 expressed digestive problems. These results suggested that the one-factor model showed a good model fit in the Chinese version of the CAS. Multigroup CFAs supported measurement invariance across genders and ages (18–29 years vs. 30 years and older), which was in line with other studies regarding measurement invariance between genders (Chen J. et al., 2021; Lieven, 2021) and between similar age groups (Lee, 2020; Ahmed et al., 2022). The measurement invariance suggested that comparing the CAS scores across genders and ages was meaningful and indicated the comparison of the CAS scores between different gender or age groups reflected the fundamental differences rather than the psychometric properties across diverse groups. Our results exhibited robust evidence of the measurement invariance across genders and ages in the Chinese sample.

The internal consistency of the CAS’ total scores (Cronbach’s alpha = 0.95, McDonald’s omega = 0.95, Spearman–Brown coefficient = 0.94) showed satisfactory internal consistency, parallel to those reported in the prior psychometric studies (Lee, 2020; Lieven, 2021; Vally and Alowais, 2021). The convergent and concurrent validity were conducted in this study. The factor loading of each item better than 0.70, AVE better than 0.50, and CR better than 0.80 supported good convergent validity of the Chinese version of the CAS and were similar to other researches using the same statistical methods (Ahmed et al., 2022; Broche-Pérez et al., 2022). Consistent with the previous studies (Choi et al., 2020; Xiong et al., 2020), higher levels of illness-specific anxiety were related to mental distress and other negative consequences. Moreover, the significant and positive associations of the Chinese version of the CAS with COVID-19-related fear and the experiences of depression, anxiety, and stress supported the concurrent validity of the CAS. The correlation between the FCV-19S and the CAS was in line with other screening pandemic-specific tools (Khosravani et al., 2021; Samimi Ardestani et al., 2022), which might indicate that COVID-19-related anxiety was triggered by exposure to coronavirus-related information (Tyrer, 2020).

Our study also had several limitations. Firstly, we conducted the online survey with self-report measurements due to the COVID-19 delta epidemic. Participants only accessed the internet to respond, resulting in the sample primarily distributed among young and middle-aged adults (*M* = 31.21, SD = 9.51). However, the elder was especially sensitive to the psycho-emotional effects of the pandemic due to the known higher risk of morbidity and mortality from COVID. It was vital to notice that the CAS Chinese version in the elderly group needed to be validated in future studies. Secondly, we used the non-random sampling method, which could lead to bias. Thirdly, this study did not analyze other reliability and validity characteristics, such as test–retest reliability and predictive validity. Evidence of test–retest reliability would be beneficial for longitudinal research in the future. Future researchers could use more objective measures to investigate the criterion validity of the CAS.

## Conclusion

In summary, the current study offered more proof in support of the psychometric properties of the CAS in different contexts as the COVID-19 pandemic develops. Previous studies supported the validation of the CAS in other cultures and diverse populations. Our study supported that the Chinese version of the CAS showed good reliability and validity during the COVID-19 delta epidemic, which proved the CAS was also valid at different times. Our research extended the research evidence on the psychometric properties of the CAS. The single-factor model had full measurement invariances across gender and age groups, which suggested that the CAS measured the same construct in different gender and age groups. Our findings supported that the CAS Chinese version was valid in assessing anxiety related to COVID-19 during the COVID-19 delta epidemic. Moreover, we translated CAS into Simplified Chinese Characters, which would provide a promising instrument for assessing anxiety related to COVID-19 in the Chinese language context.

## Data availability statement

The raw data supporting the conclusions of this article will be made available by the authors, without undue reservation.

## Ethics statement

The studies involving human participants were reviewed and approved by an ethics committee of the Second Xiangya Hospital of Central South University. The participants provided their online informed consent to participate in this study.

## Author contributions

DW conceived and designed the study. QL, LX, and DW performed the analysis and prepared the manuscript. All authors contributed to the article and approved the submitted version.

## Funding

This work was supported by the 225 High-level Health Talents Training Project of Hunan Province of China and Natural Science Foundation of Hunan Province under grant no. 2016JJ4101.

## Conflict of interest

The authors declare that the research was conducted in the absence of any commercial or financial relationships that could be construed as a potential conflict of interest.

## Publisher’s note

All claims expressed in this article are solely those of the authors and do not necessarily represent those of their affiliated organizations, or those of the publisher, the editors and the reviewers. Any product that may be evaluated in this article, or claim that may be made by its manufacturer, is not guaranteed or endorsed by the publisher.
